# Factors associated with malnutrition among pregnant women and lactating mothers in Miesso Health Center, Ethiopia

**DOI:** 10.18332/ejm/110131

**Published:** 2019-07-22

**Authors:** Masresha Leta Serbesa, Maleda Tefera Iffa, Mohammed Geleto

**Affiliations:** 1 Department of Midwifery, Harar Health Science College, Harar, Ethiopia; 2 Department of Nursing, Haramaya University, Harar, Ethiopia; 3 Miesso Health Center, Oromia Region, Ethiopia

**Keywords:** magnitude, pregnancy, malnutrition

## Abstract

**INTRODUCTION:**

Malnutrition is one of the major problems in which the physical function of an individual is impaired to the point that it can no longer maintain adequate body processes such as growth, physical work, and resistance to or recovery from disease. Malnutrition is associated with a low economic situation, and poor personal and environmental hygiene. Recent studies found that the centre of the problem is the backward socioeconomic development of the country. The level of the healthcare services in Ethiopia is low, even when compared to sub-Sahara African countries. The objective of this study was to assess the magnitude of malnutrition and associated factors among pregnant women and lactating mothers in the Miesso Health Centre, Miesso Woreda, Oromia Region, Ethiopia.

**METHODS:**

A cross-sectional study was conducted from 1 February to 30 May 2017, among pregnant women and lactating mothers in Miesso Woreda. A sample of 304 women was selected using a systematic random sampling approach from the list of patients, with different sociodemographic status.

**RESULTS:**

Our results showed that among all pregnant women and lactating mothers, 12.6% were overweight and 30.3% were underweight. From multiple logistic regression analysis, family incomes (AOR=2.056, 95% CI: 1.051–4.023) and age of women (AOR=2.169, 95% CI: 1.015–4.634) were significantly associated with the nutritional status of the study participants.

**CONCLUSIONS:**

We recommend that authorities should: facilitate the rural-urban community’s access to information on nutrition including eating-practices sanitation; initiate a health and development program during pregnancy and lactation; expand women’s education on diet during pregnancy and lactation.

## INTRODUCTION

The World Health Organization (WHO) defines malnutrition as ‘the cellular imbalance between the supply of nutrients and energy and the body’s demand for them to ensure growth, maintenance, and specific functions’. Contrary to the common use, the term malnutrition refers not only to deficiency states but also to excess and imbalance in the intake of calories, proteins and/or other nutrients^1^.

A balanced amount of nutrients is necessary for the proper functioning of the body system. Nutrition is a fundamental pillar of human life, health and development throughout the entire life span^1^. Proper food and good nutrition are essential for survival, physical growth, mental development, performance and productivity, health, and wellbeing. However, nutrition requirements vary with age, gender, and during physiological changes such as pregnancy. Pregnancy is such a critical phase in a woman's life when the expecting mother needs optimal nutrients of superior quality to support the developing fetus.

Malnutrition manifests itself as a function of many and complex factors that affect the national child status^2^. It is directly linked to inadequacy in diet and diseases under living conditions factors that include crisis in household food supply, inappropriate childcare and feeding practices, unhealthy place of residence and insufficient basic health services for those in poor socioeconomic situations, cultural beliefs, and lack of parents’ education, especially that of mothers.

An adequate nutritional status of pregnant women is essential for their health and pregnancy outcomes. Due to increased nutritional requirements, pregnancy is a critical period for meeting the body’s demand for macro/micronutrients. Thus, anaemia and vitamin A deficiency (VAD) are common micronutrient deficiencies that affect 53.8 million pregnant women in the world^3^.

Poor nutrition in pregnancy, in combination with infections, is a common cause of maternal and infant mortality and morbidity, low birth weight and intrauterine growth retardation (IUGR). Malnutrition remains one of the world’s highest priority health issues, not only because its effects are so widespread and long lasting but also because it can be eradicated best at the preventive stage^4^. Maternal malnutrition is influenced not only by lack of adequate nutrition but also influenced by social and psychological factors, nutritional knowledge of mothers, and biological changes that influence perceptions of eating patterns during pregnancies^5^.

Many women in Africa suffer from chronic energy deficiency, inadequate weight gain during pregnancy, and poor micronutrient status. Insufficient food intake, high energy expenditure, micronutrient-deficient diets, infections, and the demands of pregnancy and lactation contribute to maternal malnutrition^6^.

Maternal mortality is unacceptably high. About 800 women die from pregnancy- or childbirth-related complications around the world every day^7^. In 2013, 289000 women died during and following pregnancy and childbirth. Almost all of these deaths occurred in low-resource settings, and almost all maternal deaths (99%) occur in developing countries. More than half of these deaths occur in sub-Saharan Africa^7^.

Twenty per cent of maternal deaths in Africa have been attributed to anaemia^8^. In sub-Saharan Africa, iron and folate deficiencies are the most common causes of anaemia in pregnant women. Anaemia has a variety of converging contributing factors, but iron deficiency is the cause of 75% of anaemia cases. In Ethiopia, antenatal care (ANC) coverage by a skilled provider in 2011 was 34%. Prevalence of anaemia among pregnant women was 22%, but only 16.8% of pregnant mothers had taken iron tablets during pregnancy^9^.

Based on the above, the present study aims to assess the factors associated with malnutrition among pregnant women and lactating mothers attending antenatal care (ANC) clinics in Meisso Health Centre, Ethiopia.

## METHODS

A cross-sectional study was conducted in Miesso Health Centre, Miesso Woreda, Oromia Region, Ethiopia, from 1 to 30 March 2017. The sample consisted of 304 randomly selected pregnant women and lactating mothers.

Sample size was determined by: using a single proportion sample size calculation formula with a source of population size greater than 10000; using a 41% prevalence of malnutrition among pregnant women and lactating mothers, from previous studies done in Ethiopia; and setting the margin of error at 5% with 95% confidence interval, and a non-response rate of 10%.

Western Hararghe is the fifth largest zone of the administrative zones of Oromia Regional State. It constitutes about 7% of the size of the region and covers 22623 km^2^. The population and housing census on 2008 indicate that the Zone was projected to have a population of 2739390 (Central Statistical Authority, CSA, 2008). Miesso Woreda has a population of 175313 and about 93604 are rural inhabitants (CSA, 2008). Health service coverage of the region is 85% with four public health centers and about 23 primary health posts, with 162 health care providers, in the Miesso Woreda, but no hospital.

A structured questionnaire, containing close-ended questions including information on malnutrition and related factors such as measurement of weight, height and calculation of BMI (body mass index, kg/m^2^). The questionnaire was prepared in English and translated to Afan Oromo, a local language. Before data collection, the questionnaire was pre-tested on 5% of the patients, at a public health center in Oromia Region, who were not selected for the study but randomly selected from patients having a follow-up in the ANC clinic. In order to confirm the ethical and legal standards of the investigation, approval was obtained from the ethical review board of the Harar Health Science College. The survey was commenced after written consent was obtained from the Miesso Health Center. This helps to test the consistency and acceptability of the questionnaire. After finalizing the questionnaire by performing some necessary corrections, we trained the data collectors for 3 days. During data collection, the questionnaire was checked for completeness on a daily basis by the data collectors and the supervisors. The completed questionnaire was also rechecked by the principal investigators to maintain the quality of data.

After data collection, each questionnaire was checked for completeness, then coded and entered into Epi-info version 3.5.1 for cleaning, editing, and analysis. The results were presented in the form of tables, figures and text using frequencies and summary statistics such as mean, mode, standard deviation and percentage, to describe the study population in relation to relevant variables.

## RESULTS

The sociodemographic profile of the sample is presented in Table 1. Pregnant women were more than lactating mothers (55% vs 45%). Most of the women were aged 20–30 years, lived in rural areas, had primary school education or were illiterate, and Muslim. The majority worked as farmers with a monthly income of >1000 ETB (Ethiopian birr) (US$1 is about 29 ETB). Additionally, more than half drank unprotected water and almost half of them had access to a toilet.

**Table 1:** Sociodemographic characteristics of pregnant women and lactating mothers in Miesso Health Center (N=304)

*Respondents*	*Frequency*	*Percentage %*
Pregnant women	166	55
Lactating mothers	138	45
***Characteristics***	***Categories***	***Frequency***
Residence	Urban	178
	Rural	126
Age (years)	≤20	38
	20–30	113
	31–40	89
	41–49	64
Educational status (grades)	Primary (1–8)	150
	Secondary (9–12)	27
	Illiterate	127
Religion	Orthodox	65
	Muslim	217
	Protestant	22
Occupation	Farmer	127
	Merchant	82
	Civil servant	59
	Other	36
Family members	<4	216
	≥4	88
Monthly income ETB^a^	≤1000	143
	1000–2000	69
	>2000	92
Latrine utilization	Yes	178
	No	126
Source of drinking water	Pipe water	178
	Unprotected water	209

a Exchange: US$1 is about 29 ETB (Ethiopian Birr).

Table 2 shows the anthropometric measurements of pregnant women and lactating mothers in Miesso Health Center. According to the results, most of the women have a normal BMI while 30.3% are underweight.

**Table 2:** Anthropometric measurements of pregnant women and lactating mothers in Miesso Health Center (N=304)

*Measurements*	*Frequency*	*Median*	*Range*
Weight (kg)	124	40.8	51.5 – 60.0
	180	59.2	60.0 – 77.5
Height (m)	176	1.58	1.56 – 1.60
	128	1.66	1.61 – 1.78
	***Categories***	***Frequency***	***Percentage***
BMI (kg/m^2^)	Overweight >25.0	38	12.5
	Underweight <18.5	92	30.3
	Normal	174	57.2

Table 3 presents the nutritional status of the pregnant women and lactating mothers that participated in the study, in Miesso Health Center. The majority (83.38%) of the women have knowledge regarding their nutritional status while 67.1% are vegetarian and 22% eat meat and dairy products. Most of them eat 2–3 times within 24 hours as they follow no specific dietary regimen. Most of them (77.3%) use iodize salt to cook, do not eat fruit and vegetables (85.2% vs 68.8%, respectively), do not use iron supplements during pregnancy or lactating period (75.0%), and they do not consume more carbohydrates.

**Table 3:** Nutritional status of the pregnant women and lactating mothers in Miesso Health Center (N=304)

	*Categories*	*Frequency*	*Percentage %*
Knowledge of nutritional status	Yes	51	16.62
	No	253	83.38
Dietary style	Vegetarian	204	67.1
	Meat and dairy products	67	22
	Both equally	33	10.9
Meals within 24 hours	>3	78	25.7
	3	130	42.8
	2	96	31.6
Following specific dietary regimen	Yes	56	18.42
	No	248	81.58
Using iodized salt to cook main meal	Yes	235	77.3
	No	69	22.7
Habit of consuming fresh citrus fruits/juices	Yes	45	14.8
	No	259	85.2
Eating fruits and vegetables	Yes	95	31.3
	No	209	68.8
Iron supplements	Yes	76	25
	No	228	75
Frequency of more than usual meals per day	Yes	83	27.30
	No	221	72.70
Habit of eating more carbohydrates	Yes	73	24
	No	231	76
Eating protein, e.g. meat, milk and milk products daily	Yes	129	42.4
	No	175	57.6

Table 4 presents the health practices of pregnant women and lactating mothers who participated in the study. As shown, most of them visit the health centre often (87.2%), they have not utilized family planning (92.4%) and they give birth within a year from the last delivery (71.1%). The majority of the participants report that they receive good care (55.6%), although 72.0% do not use additional food while pregnant/lactating.

**Table 4:** Healthcare practices of pregnant women and lactating mothers in Meisso Health Centre (N=304)

*Variables*	*Categories*	*Frequency*	*Percentage %*
Often visits the health centre	Yes	265	87.2
	No	39	12.8
Family planning utilization	Yes	23	7.6
	No	281	92.4
Methods of family planning	Pills	6	2
	Injection	17	5.6
Care of pregnant women and lactating mothers	Good	169	55.6
	None	135	44.4
Birth interval (years) from last delivery	1	216	71.1
	2	88	28.9
Use of additional food for pregnant women/lactating mothers	Yes	85	28
	No	219	72

After bivariate analysis, multivariate analysis of logistic regression was performed to filter the net effect of each independent variable associated in the bivariate model analysis with nutritional status of pregnant women and lactating mothers, by controlling for the other independent variables. In a multivariate logistic regression analysis (Table 5), family income and age were found to have significant association with nutritional status of pregnant women and lactating mothers. Concerning family income, the pregnant women and lactating mothers who had a monthly income greater than 2000 ETB were less likely to be underweight than those who earned less than 1000 ETB (AOR=2.056, 95% CI: 1.051–4.023). Those mothers aged 21–30 years were less likely to be underweight than mothers older than 30 years (AOR=2.172, 95% CI: 0.843–5.598)

**Table 5:** Multivariate logistic regression of nutritional status of pregnant women and lactating mothers (N=304)

*Variable*	*Categories*	*BMI (kg/m^2^)*		*p*	*AOR (95% CI)*
		*Underweight (<18.5) n (%)*	*Normal (≥18.5) n (%)*		
Monthly income	≤1000	73 (79.35)	71 (33.49)	0.035	2.056 (1.051–4.023)
	1001–2000	17 (18.47)	51 (24.05)	0.001	5.632 (2.694–11.777)
	>2000 ETB^a^	2 (2.17)	90 (42.46)		
	Total	92	212		
Age	≤20	12 (13)	26 (12.26)		
	21–30	19 (20.7)	94 (44.34)	0.108	2.172 (0.843–5.598)
	31–40	40 (43.5)	49 (23.12)	0.046	2.169 (1.015–4.634)
	41–49 years	21 (22.8)	43 (20.28)	0.638	1.474 (0.665–3.267)
	Total	92	212		

a Exchange: US$1 is about 29 ETB (Ethiopian Birr).

## DISCUSSION

This study reports on the magnitude of malnutrition among pregnant women and lactating mothers, in Miesso Health Centre, and gives the association of sociodemographic characteristics and nutritional status of 304 participants. The study is a reasonable reflection of the nutritional status of the pregnant women and lactating mothers. A number of similar studies have been conducted in various parts of other countries.

Different studies show that education plays a prominent role on the nutritional status of pregnant women and lactating mothers. The majority of the respondents in our study were illiterate or had primary education, which may explain the diet they followed at the time of our study. Family education affects nutrition and is associated with a higher income and the participant’s ability to make better decisions for herself and her child. The educated pregnant women and lactating mothers were more careful about what they ate than the uneducated ones. A study in the urban slums of Dibrugarah showed that the prevalence of malnutrition was much higher among low income and illiterate women. These findings agree with ours.

According to the findings of our study, families with many members is also an important factor related to the nutritional status of pregnant women and lactating mothers. The reason is that there is probably not enough time for proper care and enough food for them, increasing the risk of malnutrition. The effect of large family size with overcrowding and inadequate spacing has been implicated as a risk factor for the prevalence of malnutrition.

The results of this study also show that pregnant women and lactating mothers with malnutrition live in a household with low monthly family income.

Monthly income and attitude during pregnancy were identified as important predictors of knowledge of women on nutrition during pregnancy and lactation among the study participants. A similar study conducted in Wollega showed that educational level and monthly income were significantly associated with maternal knowledge on nutrition^4^.

According to the responses on dietary style, a few were drinking fresh citrus fruit juice, almost a quarter were taking protein daily, and many were using milk and milk products daily, while a quarter were eating fresh vegetables, daily.

The weight and height of the pregnant women and lactating mothers were also measured in the study and the BMI was calculated. In all, 22.5% were overweight, 30.3% were underweight, and 47.2% of the pregnant women and lactating mothers had a normal nutritional intake. A study conducted in the rural community of the Tigray region indicated that about 25.5% and 58.3% were stunted or thin, respectively. Similarly, a study in the Jimma Zone to identify socioeconomic factors associated with underweight and stunting among adolescents found that most of the underweight adolescents (53.20%) were females. According to the 2011 Demographic and Health Survey of Ethiopia (EDHS, 2011), about 27% of women aged 15–49 years are thin, 9% are moderately or severely thin, and only 6% are overweight or obese.

## CONCLUSIONS

Education is one of the most important resources that enables the family to provide appropriate care for pregnant women and lactating mothers in terms of growth and development. The risks are increased when the monthly income is lower than 1000 ETB. According to our results, the large family size seen in the area was one of the factors affecting nutritional status. We found evidence that socioeconomic and demographic variables have a significant influence on the nutritional status of pregnant women and lactating mothers. Therefore, further actions are needed to address these problems and the implementation of effective strategies.

Based on the result of our study, we recommend that authorities should:

- Facilitate rural-urban community’s access to information on nutrition such as eating-practices sanitation, health, and development programs.
- Make efforts to expand awareness of women’s education on dietary issues during pregnancy and lactation.
- Develop educational programs about nutritional problem identification and solutions, especially on community education about specific nutritional needs of pregnant women and lactating mothers, and how to combat the main infectious diseases.
